# The Doyen of the Medical Profession at Cardiff

**Published:** 1915-04-24

**Authors:** 


### The Doyen of the Medical Profession at Cardiff.

The death has occurred of Dr. W. T. Edwards, J.P., the doyen of the medical profession at Cardiff. He was born in 1821, and for sixty years filled a large place in the public life of the city. Dr. Edwards studied medicine in London, and took his M.B. degree in 1844, distinguishing himself at the University College, London, by taking the gold medal in four subjects. In 1850 he obtained the London M.D., and in 1879 became a Fellow of the Royal College of Surgeons. By virtue of his successes the University College admitted him as a life governor. For nearly sixty years he was connected with King Edward VII.'s Hospital, Cardiff, ultimately holding in turn the positions of consulting surgeon and consulting physician, and, in addition, contributing generously to its funds. He accomplished much work for the University College of South Wales and Monmouthshire, and was a pioneer of the movement which led to the establishment of the Cardiff Medical School. He gave over £3,000 to the college for various purposes. Dr. Edwards was a vice-president of the Cardiff Liberal Association and an ardent Congregationalist.

